# Accidental Penile Trauma Resulting in a Fracture: A Case Report

**DOI:** 10.7759/cureus.85860

**Published:** 2025-06-12

**Authors:** Syed Mustafa Haider, Muhammad Saad Khan, Muhammad Amir, Ali Raza Zaidi

**Affiliations:** 1 Department of General Surgery, Surgical Unit 2, Sheikh Zayed Medical College and Hospital, Rahim Yar Khan, PAK

**Keywords:** eggplant deformity, foley catheter, penile fracture, penile trauma, primary surgical repair, sexual intercourse, urethral injury

## Abstract

A penile fracture is an uncommon but urgent urological emergency resulting from the rupture of the tunica albuginea, typically during vigorous sexual activity or trauma to the erected penis. It commonly presents with acute pain, rapid detumescence, and the characteristic “eggplant deformity.” Prompt diagnosis and surgical intervention are essential to prevent long-term complications such as erectile dysfunction, urethral injury, and penile curvature. We report a rare presentation of a penile fracture in a 38-year-old male who experienced sudden localized penile swelling following accidental trauma without attempted intercourse. Notably, classical symptoms such as severe pain, bruising, and a snapping sound were absent. On examination, localized swelling was present without discoloration or per-urethral bleeding. The defect in the corpora was successfully repaired using absorbable sutures following subcoronal incision. Postoperative recovery was uneventful, and the patient was discharged on oral antibiotics with counseling regarding sexual abstinence. This case highlights the importance of maintaining high clinical suspicion for a penile fracture, even in the absence of classical signs. Early surgical management remains the standard of care to ensure favorable outcomes. In addition, this report emphasizes the need for individualized assessment, as atypical presentations may delay diagnosis and treatment. Awareness and timely intervention are critical to minimize the risk of chronic complications and preserve sexual function.

## Introduction

A penile fracture refers to injury to the tunica albuginea in the form of a tear or abruption, most commonly after engaging in strenuous sexual activities. Penile trauma can also result from overbending during erection, excessive masturbation, accidental blunt injury, or complications from Peyronie’s treatment with collagenase *Clostridium histolyticum* [1]. A penile fracture is often marked by an audible cracking sound, acute pain, sudden detumescence, and visible swelling and discoloration of the penile shaft, commonly referred to as the "eggplant deformity." Diagnosis is typically made through a combination of patient history and physical findings. A penile fracture most commonly presents with pain (35.7%), hematoma (29.8%), and edema (11.9%). The majority of cases involve the right corpus cavernosum, with an average delay of 5.9 hours before seeking medical care [2].

A penile fracture warrants immediate surgical intervention. In cases of partial urethral injury, management options include urethral catheterization, primary repair with nonabsorbable sutures, or suprapubic diversion. Prompt surgical treatment significantly minimizes long-term complications [3].

Delayed surgical intervention or reliance on conservative management in cases of a penile fracture is associated with a heightened risk of missed urethral injuries and subsequent complications, including urethral stricture, fistula, penile abscess, curvature, persistent hematoma, and erectile dysfunction. Postoperative complications occur in approximately 4-12% of cases. The standard postoperative protocol advises a six-week period of sexual abstinence, with re-initiation of sexual activity dependent upon the full resolution of local postoperative symptoms, including pain, edema, and ecchymosis [4].

## Case presentation

**Figure 1:**
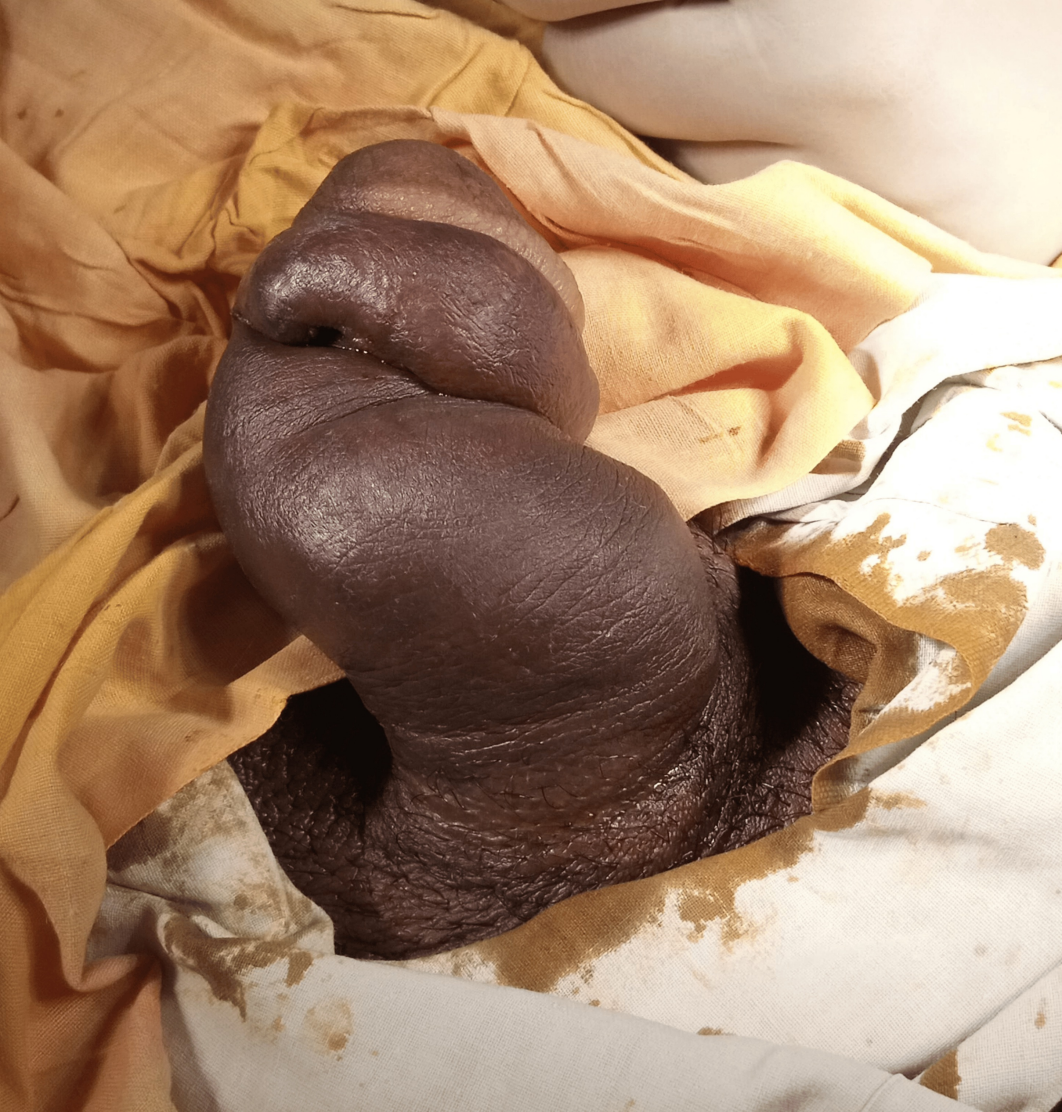
Gross deformity of the penis It should be noted that there is no bruising, and the figure does not depict classical “eggplant” deformity.

We report a 38-year-old male who presented to the emergency department with the complaint of swelling of his penile shaft. The swelling was sudden in onset, starting within a few seconds following trauma to the penis. There was also a brief episode of pain, which lasted for less than an hour. The pain was described as 4/10 by the patient on a visual analogue scale. The swelling progressed over the course of one minute. After that, no increase in swelling was noted by the patient. The patient did not plan insertion into the vagina, and the patient described the incident as an accident. The patient came to the emergency department after 12 hours. Following the incident, swelling remained confine to the shaft of the penis (Figure 1).

He reported experiencing mild, localized, dull pain immediately after the incident, which resolved spontaneously prior to his arrival at the hospital. He was able to urinate without difficulty and denied any hematuria, supra pubic discomfort, or abdominal pain.

Upon arrival at the emergency department, a clinical diagnosis of a penile fracture was promptly made. On presentation, there was no blood at the tip of the urethral meatus.

The patient was managed with intravenous analgesics and empirical antibiotics. A perioperative evaluation was conducted, and the patient was deemed fit for spinal anesthesia. His medical history revealed early-onset type 2 diabetes mellitus, managed with dual-acting insulin. Notably, there were no diabetes-related complications, including erectile dysfunction.

**Figure 2:**
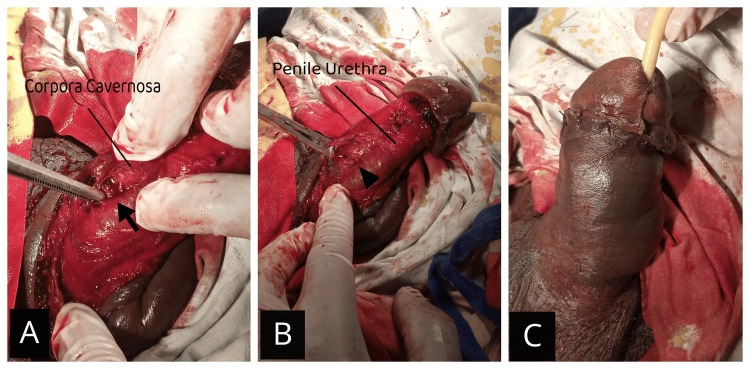
Images showing the 1 cm defect in the corpora, on the right lateral side (intraoperatively) The defect is pointed by plain forceps, representing the tear in the corpora covernosum, following the bending of the penile shaft, as shown by the arrow (A). Closure of the defect by absorbable suture, with foleys catheter in situ, pointed by arrow head (B). Penile shaft after closing the skin and subcutaneous tissue (C).

In the operating room under spinal anesthesia, a subcoronal incision was made. A careful exploration revealed a 1 cm rent in the right corpus cavernosum, about 2 cm from the base, along with a few hematomas (Figure 2).

The continuity of the urethra was confirmed, and a 16 Fr Foley's catheter was successfully inserted. The defect was closed using 3-0 Vicryl, while the skin and subcutaneous tissue were approximated with 2-0 Vicryl. There was minimal intraoperative blood loss with no complications.

The patient was shifted to the surgical ward. The recovery was uneventful, and the Foley's catheter was removed on the third postoperative day. He was discharged with a prescription for oral antibiotics and analgesics. Bicalutamide 150 mg/day was prescribed for six weeks to prevent penile erection, which may lead to a recurrent penile fracture. The patient was counseled regarding abstinence from sexual activity for six to eight weeks. On follow-up at three months, no penile deformity, erectile dysfunction, or dyspareunia was observed.

## Discussion

A penile fracture is an infrequent emergency, mostly presenting in middle-aged men. It usually follows traumatic injury to the penis while sexual intercourse, at insertion [5,6]. Since the first case report of a penile fracture in the literature in 1924, hundreds of cases have been reported from all over the world [6]. The maximum number of cases has been reported in a study conducted on the Iranian population, with 987 cases over a period of three years [2]. However, no data on the incidence of penile fractures in Pakistan is available. A retrospective case series conducted from 1997 to 2007 included 12 patients with penile fractures [5].

The tunica albuginea, classically regarded as the toughest fascia in the human body, has the tensile strength of about 104 to 105 N/m2 and a thickness of about 2 mm in the flaccid state. During erection, the thickness is reduced, which, together with its tensile strength, makes it vulnerable to fractures easily during bending and traumatic injury, as in our case [5-7].

Commonly reported symptoms are pain and swelling, extending to the scrotum, along with bruising of the penis [1-4]. The classical ‘‘popping’’ associated with the penile fracture and bruising of the penis were not present in our case [4-6]. Absorbable suture (Vicryl 3.0) was used to repair the defect, possibly due to the formation of painful and palpable knots with non-absorbable suture, postoperatively [5].

Bicalutamide, a non-steroidal androgen receptor antagonist, was prescribed to suppress erections, with the latest literature supporting its use [8]. Retrograde urethrography was also not performed, due to the history of passage of urine by the patient, following the incident. Moreover, Foley's catheter insertion perioperatively did not show any sign of urethral involvement [2,3].

In suspected cases of penile fractures, regardless of the etiology, surgery is considered the treatment of choice. Immediate exploration is undertaken to reduce the long-term complications, such as impotence, urethral stricture, and pain during sexual intercourse [6-9].

## Conclusions

This case suggests that patients with penile fractures do not always have classical signs and symptoms. Prompt decision-making and surgical exploration should be undertaken to reduce long-term morbidity associated with the condition. Concomitant urethral injury must be ruled out by history, clinical examination, and investigations (retrograde urethrography and magnetic resonance imaging).
